# Overcoming failure: improving acceptance and success of implanted neural interfaces

**DOI:** 10.1186/s42234-025-00168-7

**Published:** 2025-03-14

**Authors:** Ashley N. Dalrymple, Sonny T. Jones, James B. Fallon, Robert K. Shepherd, Douglas J. Weber

**Affiliations:** 1 Department of Biomedical Engineering, University of Utah, Salt Lake City, UT USA; 2 Department of Physical Medicine and Rehabilitation, University of Utah, Salt Lake City, UT USA; 3 NERVES Lab, University of Utah, Salt Lake City, UT USA; 4 Department of Mechanical Engineering, Carnegie Mellon University, Pittsburgh, PA USA; 5 NeuroMechatronics Lab, Carnegie Mellon University, Pittsburgh, PA USA; 6 Bionics Institute, St. Vincent’s Hospital, Melbourne, VIC Australia; 7 Medical Bionics Department, University of Melbourne, Melbourne, VIC Australia; 8 Neuroscience Institute, Carnegie Mellon University, Pittsburgh, PA USA

**Keywords:** Neural interfaces, Biocompatibility, Medical devices, Chronic in vivo, Bioelectronics, Failure testing

## Abstract

Implanted neural interfaces are electronic devices that stimulate or record from neurons with the purpose of improving the quality of life of people who suffer from neural injury or disease. Devices have been designed to interact with neurons throughout the body to treat a growing variety of conditions. The development and use of implanted neural interfaces is increasing steadily and has shown great success, with implants lasting for years to decades and improving the health and quality of life of many patient populations. Despite these successes, implanted neural interfaces face a multitude of challenges to remain effective for the lifetime of their users. The devices are comprised of several electronic and mechanical components that each may be susceptible to failure. Furthermore, implanted neural interfaces, like any foreign body, will evoke an immune response. The immune response will differ for implants in the central nervous system and peripheral nervous system, as well as over time, ultimately resulting in encapsulation of the device. This review describes the challenges faced by developers of neural interface systems, particularly devices already in use in humans. The mechanical and technological failure modes of each component of an implant system is described. The acute and chronic reactions to devices in the peripheral and central nervous system and how they affect system performance are depicted. Further, physical challenges such as micro and macro movements are reviewed. The clinical implications of device failures are summarized and a guide for determining the severity of complication was developed and provided. Common methods to diagnose and examine mechanical, technological, and biological failure modes at various stages of development and testing are outlined, with an emphasis on chronic in vivo characterization of implant systems. Finally, this review concludes with an overview of some of the innovative solutions developed to reduce or resolve the challenges faced by implanted neural interface systems.

## Background

**Fig. 1:**
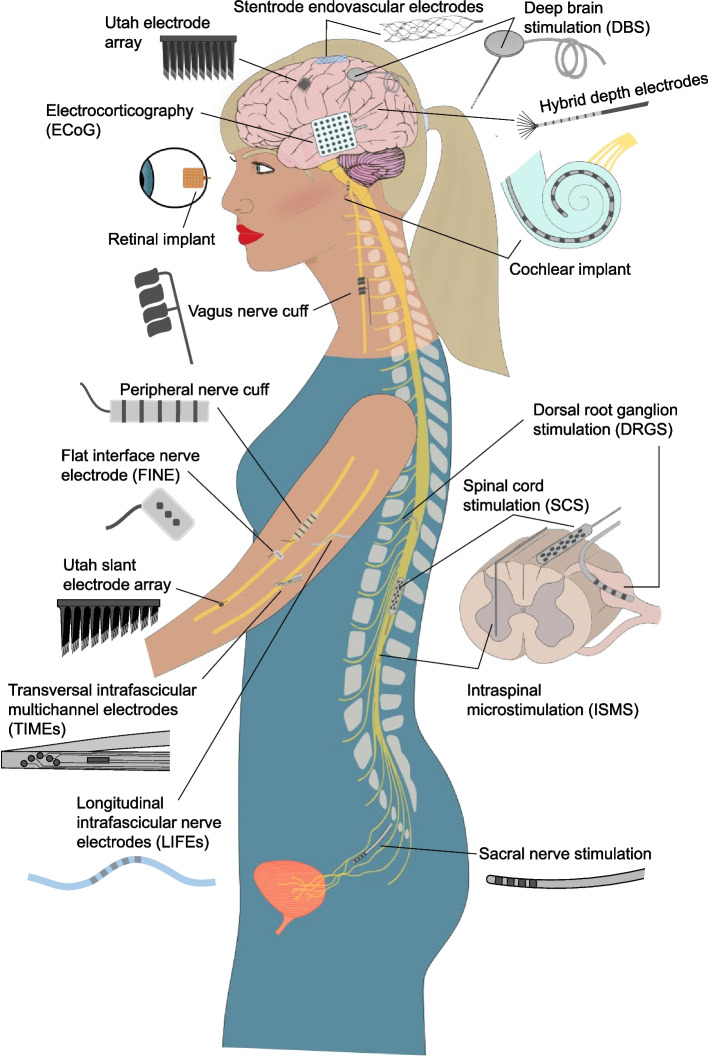
Illustration of neural interfaces that have been implanted in humans

**Fig. 2:**
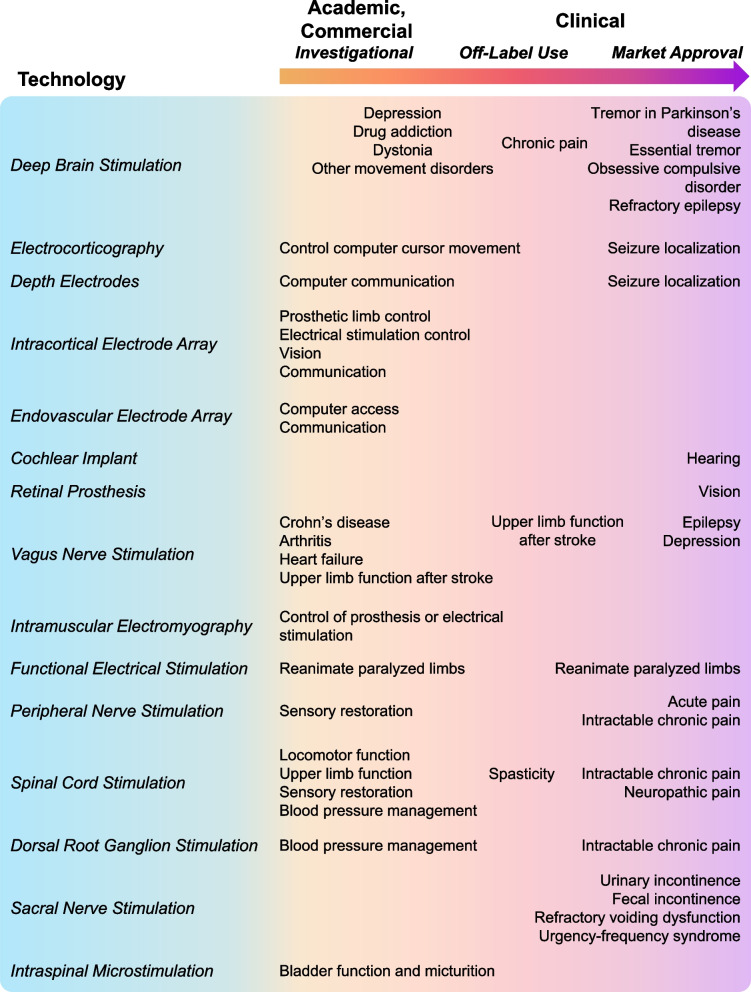
Examples of implantable neural interfaces that have been implanted in humans. Some of these technologies have market approval for clinical use, some are used clinically off-label, while many others are under investigation in academia and in industry and are limited to clinical trials. References: Deep brain stimulation: (Drobisz and Damborská, 2019; Kalia et al. 2013; Kogan et al. 2019; Li and Cook 2018; Rapinesi et al. 2019; Wang et al. 2018); Electrocorticography: (Berger and Ojemann 1992; Leuthardt et al. 2004); Depth electrodes: (Krusienski and Shih 2011; Lehongre et al. 2022); Intracortical electrode array: (Ajiboye et al. 2017; Barry et al. 2023; Collinger et al. 2013; Deo et al. 2024; Drew 2024; Flesher et al. 2021; Hosman et al. 2023; Rastogi et al. 2021; Willett et al. 2023, 2021); Endovascular electrode array: (Mitchell et al. 2023; Oxley et al. 2020); Epidural spinal cord stimulation (Angeli et al. 2018; Barolat et al. 1988; Bose et al. 2025, 2024; Capogrosso et al. 2024; Carhart et al. 2004; Chandrasekaran et al. 2020; Darrow et al. 2019; Dekopov et al. 2015; Gill et al. 2018; Goodwin et al. 2023; Harkema et al. 2018; Iversen et al. 2024; Nanivadekar et al. 2023; Pinter et al. 2000; Powell et al. 2022; Raslan et al. 2007; Richardson and McLone 1978; Shealy et al. 1967; Singh et al. 2023; Squair et al. 2021; Tator et al. 2012; Wagner et al. 2018); Cochlear implant: (Shepherd et al. 2013; Zeng et al. 2008); Retinal prosthesis: (Ayton et al. 2014; Christie et al. 2022a; Gregori et al. 2016); Vagus nerve stimulation (Austelle et al. 2022; Dawson et al. 2021; De Ferrari et al. 2011; Dibué-Adjei et al. 2019; Koopman et al. 2016; Kosel et al. 2011; LivaNova 2023; Rush et al. 2005; Sinniger et al. 2020); Dorsal root ganglion stimulation: (Deer et al. 2019; Liem et al. 2013; Sverrisdottir et al. 2020); Peripheral nerve stimulation: (Charkhkar et al. 2018; Gan et al. 2012; George et al. 2019; Goree et al. 2024; Raspopovic et al. 2014; Tan et al. 2014); Functional electrical stimulation: (Chaplin 1996; Hardin et al. 2007; Kobetic et al. 1999; Makowski et al. 2021; Peckham et al. 2001); Intramuscular electromyography: (Hart et al. 2011; Heald et al. 2019; Page et al. 2018); Intraspinal microstimulation: (Nashold et al. 1981, 1972); Sacral nerve stimulation (Hull et al. 2013; Sukhu et al. 2016; Tanagho et al. 1989)

**Fig. 3:**
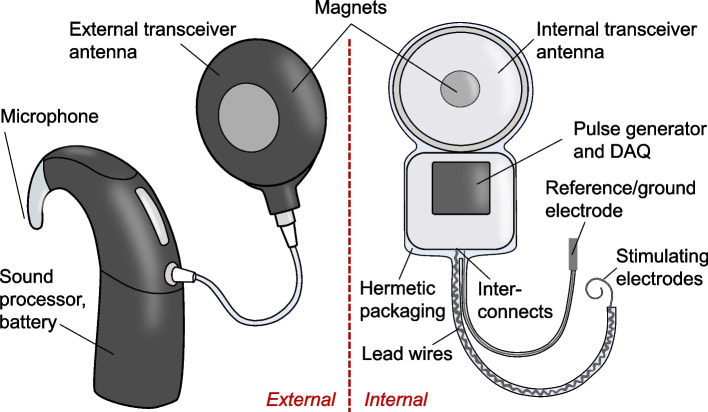
Cochlear implant, demonstrating the variety of components of implanted neural interfaces. The magnets connect the external (left) and internal (right) units across the skin. The microphone on the external unit records sound, the sound processor converts the sound recordings to a digital signal with stimulation commands, the stimulation commands and power from the battery are sent from the external to the internal transceiver, the stimulation commands are converted into a stimulation output by the pulse generator, the lead wires carry the current to the electrodes, which generate an electric field that activates auditory neurons that lie outside the cochlea. Recording of impedance at the electrode-tissue interface can also occur, where the DAQ records the electrode impedance with reference to the ground electrode, and transmits these data to a different external transceiver to transfer the data to a computer for viewing by clinical team members

Implanted neural interfaces are electronic devices that interact with the nervous system to sense (record) or stimulate neural activity to treat neurological disorders. They differ slightly from other implanted bioelectronic devices, such as pacemakers, in that they are implanted in or near neural tissue, including the brain, spinal cord, or peripheral nerves (Fig. 1). Several neural interfaces for stimulating and recording, such as cochlear implants, deep brain stimulation (DBS), spinal cord stimulation (SCS), electrocorticography (ECoG), and depth electrodes have market approval and have been used clinically for decades with great success. Some of these devices are used clinically “off-label” to explore new treatment applications, and several emerging neural interface technologies are being developed and tested in commercial and research investigational clinical trials (Fig. 2). The major components of implanted neural interface systems are: (i) the pulse generator (for stimulation) and/or data acquisition (DAQ) device (for recording), (ii) power and communication, (iii) packaging, (iv), lead wires and interconnects, and (v) electrode(s) (example of a cochlear implant system shown in Fig. 3).

Pulse generators deliver electrical current to activate the target neurons. Recording DAQ devices typically contain amplifiers and filters to increase the signal-to-noise ratio of the recorded signal. Both stimulating and recording devices might also have components for additional processing and control. While many clinical devices use fully implantable pulse generators, including SCS and DBS, other devices, such as many models of cochlear implants, employ inductive coupling to transfer power and control signals wirelessly across the skin. Furthermore, SCS and DBS implantable pulse generators sometimes require communication with external modules for data transfer or recharging.

Wireless power transmission, battery recharging, and communication to implanted neural interfaces typically uses inductive radio frequency (RF) or near-field coils on either side of tissue (for example, skin (Zeng et al. 2008) or dura mater (Powell et al. 2017)). The internal and external RF coils must be coupled by overlapping physically as much as possible with a small thickness of tissue in between; this may be difficult in some anatomical locations such as the skull or back (Troyk and Rush 2009). The more electrodes in a neural interface the higher the power and data rate transmission requirements, increasing the bandwidth and power consumption (Nair et al. 2023). Data and power transmission are limited due to safety restrictions: the power density in the body must be < 80 mW/cm^2^ to avoid tissue damage from heating (Seese et al. 1998).

Implanted electronics are packaged, typically in rigid Titanium housing (Sidambe 2014), which acts as a biocompatible hermetic seal. Hermetic seals keep the enclosed electronics safe and sound from the moisture and ions in the tissue (Merrill 2014). Wires connect the internal components to external components such as electrode leads via a feedthrough assembly that often consists of a ceramic or fused silica insulator (Nagarkar et al. 2017).

Lead wires connect the electrodes to the pulse generator and/or DAQ unit. They are insulated, often with silicone, polyimide, parylene, or other flexible inert polymer materials (Barrese et al. 2013; Kuo et al. 2013).

**Fig. 4:**
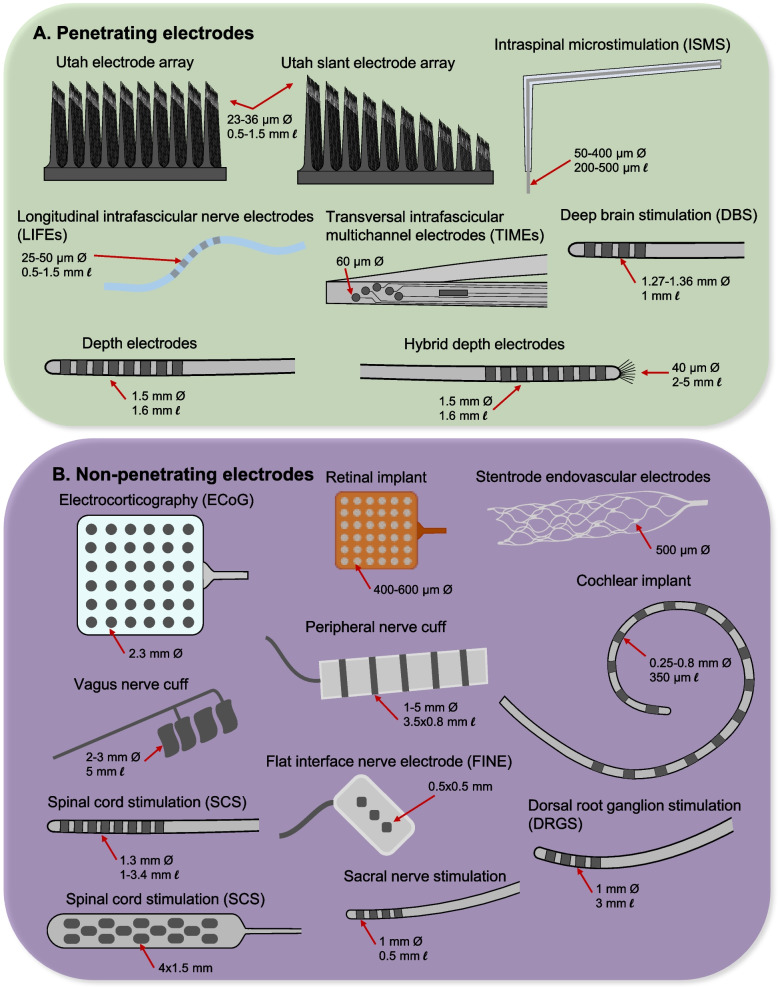
Types and dimensions of different types of implanted neural interface electrodes in humans. **A** Penetrating electrodes, **B** Non-penetrating electrodes. References: UEA: (Blackrock Neurotech 2023a; Campbell et al. 1991); USEA: (Blackrock Neurotech 2023b); ISMS: (Bamford et al. 2017; Dalrymple et al. 2018; Nashold et al. 1972); LIFE: (Malagodi et al. 1989; Rijnbeek et al. 2018); TIME: (Boretius et al. 2010); DBS: (Butson and McIntyre 2006; Medtronic 2022); Depth electrodes: (Fu and Rutishauser 2025); ECoG: (Dubey and Ray 2019); Retinal: (Ayton et al. 2014); Stentrode: (John et al. 2019); Vagus: (Mehta et al. 2018; Suminski et al. 2023); PNS cuff: (Fisher et al. 2009); Cochlear: (Dalrymple et al. 2019; Nguyen et al. 2013); SCS linear: (Boston Scientific, 2017; Medtronic 2017); SCS paddle: (Medtronic 2017); FINE: (Tyler and Durand 2002); Sacral: (Markland et al. 1972; Rijkhoff et al. 1997, 1994); DRGS: (Al-Kaisy et al. 2019)

Electrodes are the conductive materials that interface with neurons. They come in many forms including rings, pads, and shanks and can be penetrating or non-penetrating into the neural tissue (Fig. 4). Electrodes can vary greatly in size, depending on the neural target and electrode density, although electrodes on clinically available leads are more similar to each other. Some electrode designs, such as those for intraspinal microstimulation or longitudinal intrafascicular electrodes (LIFEs), use a continuous material for the lead wire and electrode, where the electrode is simply the de-insulated portion of the lead (Bamford et al. 2017; Rijnbeek et al. 2018). Depth electrodes are similar in design to many clinically-available implanted electrodes; however, they come in a hybrid version where microwires splay from the tip of the array to record from single neurons (Fu and Rutishauser 2025). Most clinical electrodes are comprised of platinum or platinum-iridium alloys (Cogan 2008; Ford 2010; Stöver and Lenarz 2011). Pad-type electrodes, including those used for transverse intrafascicular multichannel electrodes (TIMEs) and ECoG, use sputtering, etching, stamping, or welding manufacturing techniques (Boretius et al. 2010; Konerding et al. 2018). Many other electrodes, such as cylindrical electrodes or those used for intracortical interfaces, require metal bonding or welding (Barrese et al. 2013). Coatings on the electrodes are sometimes used to increase the conductivity; commonly, iridium oxide is used (Woeppel et al. 2021).

Many implanted neural interfaces remain effective in humans for years to decades (Table 1). Despite the great successes of implanted neural interface systems, each of the aforementioned components is a potential source of failure. Furthermore, each application has specific challenges, such as surgical access, safe implantation, movement of local tissues, and electrochemical reactions at the electrode-tissue interface. The effectiveness and reliability of neural interfaces are limited by these technological and biological challenges that can impede translation and years-long use of the devices in humans. In the following sections, we will review several of these barriers and how they affect device longevity, with a focus on devices that have been implanted in humans. We will then highlight failure modes of devices during chronic in vivo implantation with specific examples reported from clinical use or clinical trials. We will also review failure modes associated with the development of novel implantable devices. With each failure mode, solutions arise to resolve the issues. We conclude with novel neural interfacing technologies and how they may be able to mitigate some of the issues experienced by traditional neural interface systems during long-term implantation.

**Table 1:** Reports of implanted device duration in humans

*Name*	*Duration Human Implants*
Deep brain stimulation (DBS)	Years to decades (Haberler et al. 2000; Moss et al. 2004)
Electrocorticography (ECoG)	Months to years (Nurse et al. 2018)
Depth electrodes	Weeks (Lehongre et al. 2022)
Intracortical electrodes	Years to decades (Hughes et al. 2021; Sponheim et al. 2021; Szymanski et al. 2021; Towle et al. 2020; Woeppel et al. 2021)
Stentrode endovascular electrode array	Years (Mitchell et al. 2023)
Spinal cord stimulation (SCS)	Years (Cameron 2004; Costandi et al. 2020)
Intraspinal microstimulation (ISMS)	> 10 years (Nashold et al. 1981)
Dorsal root ganglion stimulation (DRGS)	Years (Deer et al. 2020; Morgalla et al. 2018)
Cochlear implant	Years to decades (Kim et al. 2020; Nadol et al. 2014; O’Malley et al. 2017)
Retinal prosthesis	Months to Years (Christie et al. 2022b; Daschner et al. 2017; Muqit et al. 2019)
Vagus nerve stimulation	Years to decades (Dibué-Adjei et al. 2019; Siddiqui et al. 2010)
Peripheral nerve stimulation (penetrating)	Years (Čvančara et al. 2023; George et al. 2020)
Peripheral nerve stimulation (cuff)	Years (Christie et al. 2017; Fisher et al. 2009)
Functional electrical stimulation	Years (Kobetic et al. 1999; Triolo et al. 2018, 2012)
Sacral stimulation	> 5 years (Hull et al. 2013; Siegel et al. 2018)

## I knew you were trouble

There are many reasons for the reduced effectiveness or even failure of implanted neural interfaces, including technological, mechanical, and biological barriers (the latter literally forms a barrier). Assuming the manufacturing process was consistent and of high quality and the surgical implant was free from iatrogenic damage to the device or recipient (see Barrese et al. 2013 for examples), there are still many points of failure for implanted neural interfaces. Characterization of failure modes for the different types of neural interfaces varies. Brain-computer interfaces (BCIs) using intracortical electrodes often have a reduced ability to record from neurons over time, leading many researchers to investigate why these interfaces have a limited lifetime (Barrese et al. 2013; Chestek et al. 2011; Colachis et al. 2021; Kozai et al. 2012b; Prasad et al. 2014). However, all neural interfaces pose challenges with long-term communication with the nervous system.

### Delicate electronics

Implanted pulse generators and DAQs typically consist of a battery and electronic components. Fully implantable stimulators and recording DAQs allow the omission of transcutaneous wires, which can be a source of infection and fistula formation (DeMichele et al. 2014; Weir et al. 2009). However, this means that a revision surgery is required to replace the battery or unit for devices that do not have rechargeable batteries, or rechargeable batteries that eventually lose the ability to recharge. A recent study found that patients receiving DBS prefer fixed-life batteries compared to rechargeable because not having to recharge batteries impacts their lifestyle less (Khaleeq et al. 2019); however, this patient population is typically older and may not require revision surgery in their remaining lifetime. Another important factor to consider is that the risk of infection is higher for impulse generator replacement than the initial implant procedure (Pepper et al. 2013). Therefore, it is imperative that the electronic components are reliable and the battery lasts as long as possible.

Complex systems that require onboard control systems or signal processing will require more power, demanding more from the battery or may require a larger battery. Larger batteries will require larger housing, limiting the placement of the housing and possibly requiring longer lead wires between the housing and the electrodes. Large pulse generators and DAQ housing can also increase the risk of developing dermatitis or pressure sores that degrade the tissue surrounding the housing implant (Choi et al. 2021; Dujari and Gold 2019; Hamada et al. 2006). Severe pressure sores and wound complications such as hematoma, seroma, biofilm formation, skin erosion, and dehiscence occur in 1 – 27% of patients and can result in loss of skin over the pulse generator and even the need for explantation (Falowski et al. 2019; Hanna et al. 2024; Prabhala et al. 2023; Spindler et al. 2023; Xiao et al. 2024). Form factor and stiffness of the housing likely affect wound healing and the incidence of pressure sores; however, these factors are not reported, likely because the housing is identical to non-neural implants, including pacemakers (Clingan et al. 2020).

Notably, the largest part of the power budget is for wireless communication. Heating from data and power transmission can occur from both the RF coils and the implanted circuitry (Troyk and Rush 2009). Wireless transmission is also complicated by the desire to reduce the size of the implanted coil, as many designs seek to be compact and completely wireless, such as the floating microelectrode array for intracortical visual prostheses (Troyk et al. 2005) and peripheral nerves (Bredeson et al. 2015). These floating arrays have all electrodes, electronics, and RF coils onboard a single device. To minimize the implant fingerprint means using a small diameter (on the order of mm or smaller) implanted RF coil. The reduced size of the internal RF coil reduces the coupling of the coils, which then demands a higher intensity magnetic field and increases the power requirements (Nair et al. 2023; Troyk and Hu 2013). Power consumption needs to be considered for both external and implanted pulse generators and DAQs, as high power consumption reduces battery life and necessitates more frequent battery recharging or replacement.

Maintaining a hermetic seal with high-density feedthroughs is still a challenge facing implanted electronics. Hermetic seal failure can lead to a host of problems, including damage to electronic components, corrosion, short circuits, open circuits, current leakage, damage to or change in properties of wireless coil wires, and loss of amplifier sensitivity (Breach et al. 2010; DeMichele et al. 2013; Merrill 2014).

Lead wire damage can occur at implant, during the implant period, or upon removal. Many designs allow for bundling or coiling to provide strain relief, which is important for preventing dislodgement of the implants (Dadd et al. 2011; Greenberg et al. 2002; Marsolais and Kobetic 1986). However, lead wires are susceptible to mechanical fatigue from frequent bending, especially when the lead wires run through regions of the body subject to movement, such as the limbs (Pena et al. 2017; Phillips et al. 2004), the eyes (Ayton et al. 2014), the supraclavicular region (Mohit et al. 2004), and even the spinal cord (Toossi et al. 2017). Lead wire fractures are common in people with dystonic movement disorders (Yianni et al. 2004). Lead wire damage can induce cracks in the insulation, resulting in electrical leakage (Bredeson et al. 2013; Lyons et al. 2004; Prasad et al. 2014). Damage to lead insulation not only reduces the effectiveness of the device due to less current reaching the target tissue, but also poses a safety risk through off-target stimulation (Pena et al. 2017). Deinsulation of the lead wire can also be an issue, especially for shank electrodes (Prasad et al. 2014, 2012). Peeling of the insulation away from the tip of the electrode during insertion can increase mechanical damage to the tissue. Deinsulation also increases the surface area of the electrode, which can reduce specificity and change the expected electrochemical behaviour of the electrode. Lead wires are also susceptible to breakage, which results in an open circuit and could lead to device failure. Additionally, lead wire breakage can result in fragments remaining inside the body, as reported following the removal of sacral stimulation leads (Rueb et al. 2020).

Pad electrodes are at risk of delamination or loss of bonding from the carrier (Čvančara et al. 2020). Delamination can occur when the bond between the pad electrode has poor adhesion with the substrate or when liquid leaches in between the pad electrode and substrate (Dalrymple et al. 2019; Green et al. 2012; Prasad et al. 2014). Many electrodes, such as ring electrodes, some pad electrodes, or electrodes used for intracortical interfaces, require metal bonding or welding (Barrese et al. 2013). This junction is a potential source for discontinuity and may result in conduction failure due to an open circuit or result in current leakage. Furthermore, manufacturing differences have been identified as a source of failure for intracortical electrode arrays (Prasad et al. 2012). Deformities in the tips of the electrodes from laser cutting have occurred, as well as differences in the extent of the deinsulation of the wires, even in arrays manufactured in the same batch. These manufacturing defects led to insulation cracks and increased tissue response. Intracortical electrode arrays are quite brittle, especially those made of silicon or ceramic, and these electrodes must be handled with care during implantation (Barrese et al. 2013; Ward et al. 2009). Reducing the size of electrodes can increase specificity and reduce the implant profile in the tissue; however, smaller electrodes are more difficult to handle and are often extremely fragile. Additional challenges with small and high density electrodes are increased power and communication requirements (Troyk and Rush 2009), cross-talk, increased impedance (Nelson et al. 2017), and increased charge density during stimulation. Increased charge density can lead to focal tissue damage (discussed more below).

Electrode geometry influences the charge density during electrical stimulation. Electrodes with irregular geometries that are not spherical will have a non-uniform charge density (Bruckenstein and Miller 1970; Harnack et al. 2004). For example, electrode pads will accumulate charge on the edges (Wiley and Webster 1982), and penetrating electrodes will accumulate charge at the tip (McCreery et al. 2010). When charge is injected into the tissue through an electrode, the reactions at the electrode-tissue interface can be described as reversible or irreversible. Irreversible reactions lead to the electrolysis of water; the cathodic and anodic potentials that cause the electrolysis of water are known as the water window (Cogan 2008). Water hydrolysis results in the formation of hydrogen and oxygen gases, a pH change at the interface from hydroxyl ions, the formation of reactive oxidation species, and electrode dissolution and/or corrosion (Cogan 2008; Merrill et al. 2005; Shepherd et al. 2021). These reactions may lead to electrode failure, tissue reactivity, and necrosis. Using charge-balanced waveforms that are limited in potential according to the water window can help minimize irreversible reactions (Brummer and Turner 1977). However, focal areas of charge accumulation as a result of electrode geometry can lead to local irreversible reactions between the electrode and tissue. Furthermore, at areas of high charge density, local corrosion or tissue damage may occur (McCreery et al. 1990; Shepherd et al. 2021; Wiley and Webster 1982). Electrode corrosion is worse in the presence of reactive oxygen species (Patrick et al. 2011). Electrode corrosion can result in the dispersion of metal particulates into the surrounding tissue (Dymond et al. 1970; Patrick et al. 2011; Shepherd et al. 2021, 2020, 2019). Corrosion is not limited to stimulating electrodes. Recording electrodes can undergo corrosion due to material degradation by the foreign body tissue response (described below; Merrill 2010; Prasad et al. 2012). The presence and rate of corrosion is influenced by both the tissue environment and material. For example, Tungsten and stainless steel electrodes are more likely to corrode than Platinum-Iridium or Titanium electrodes (Cogan 2008; McCarthy et al. 2011; Patrick et al. 2011; Prasad et al. 2014, 2012). Platinum compounds, Tungsten ions, and Silver can all be cytotoxic, limiting the lifetime of the electrodes (Dymond et al. 1970; Patrick et al. 2011; Shepherd et al. 2021). However, Platinum is able to convert oxidative species to water, rendering the oxidative species inert and less dangerous (Patrick et al. 2011).

### A treacherous environment

Any foreign material implanted in the body, including biocompatible materials, will evoke a foreign body response, which can reduce the efficacy of implanted neural interfaces. Additionally, with any surgical procedure, there is always a risk of infection. Infections following device implantation can result in biofilms forming on the implant, making clinical treatment difficult and could require device removal in addition to permanent tissue damage. The foreign body response, or tissue response, is the immune system’s rejection of a foreign body. The tissue response is also influenced by the extent of the trauma from insertion; less invasive, smaller devices will evoke a smaller tissue response. The failure of intracortical electrodes to record from single neurons long-term has been largely attributed to the tissue response of the brain (Moxon et al. 2009). The tissue response to implants in the central and peripheral nervous systems involve different immune cells and can be divided grossly into acute and chronic phases. The acute phase can also be separated into acute (within 24 h) and sub-acute phases (within a few days).

#### Central nervous system tissue response

**Fig. 5:**
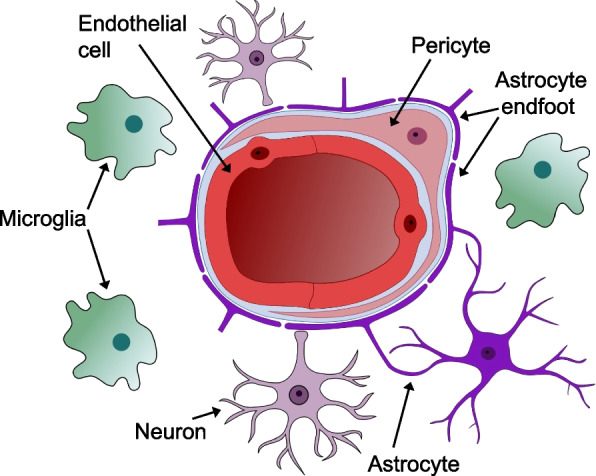
Cross-section of a blood vessel in the central nervous system, illustrating the neurovascular unit. The neurovascular unit maintains the blood–brain barrier and is comprised of endothelial cells, pericytes, microglia, astrocytes, and neurons

**Fig. 6:**
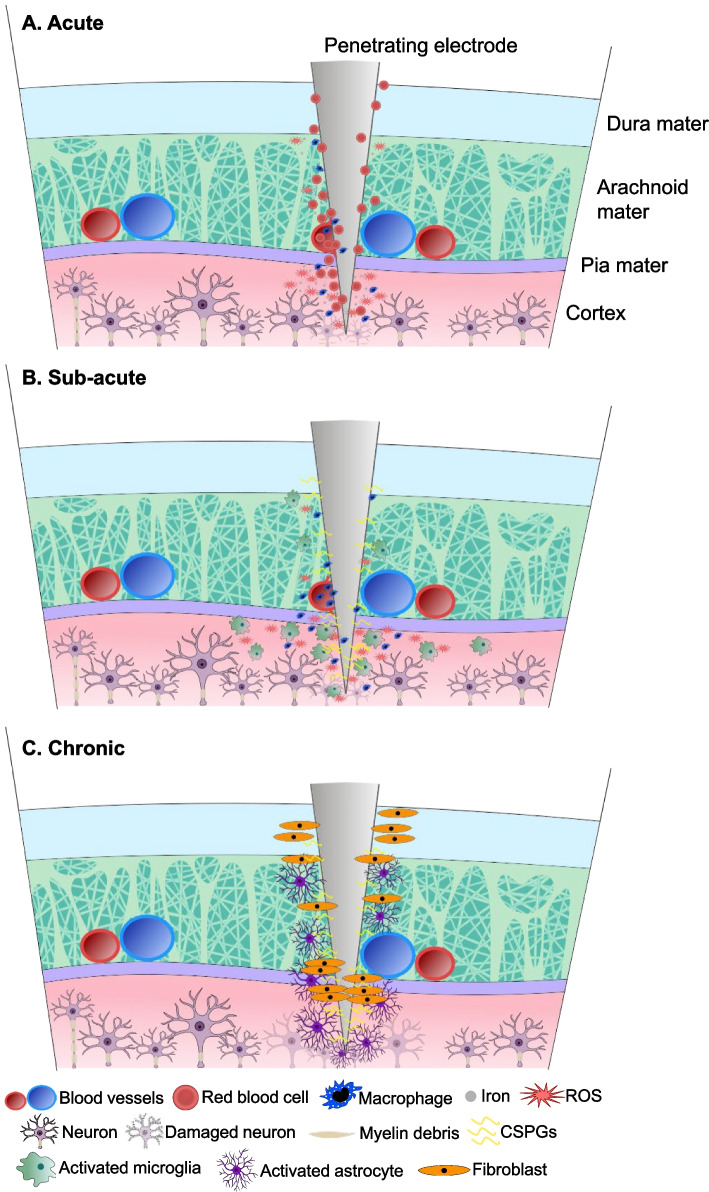
Acute, sub-acute, and chronic tissue response to implanted neural interfaces in the central nervous system

The central nervous system (CNS) includes the brain, cerebellum, brainstem, and spinal cord. The CNS is contained within the blood–brain barrier (BBB), which separates the CNS from the vascular system. The BBB is composed and maintained by the neurovascular unit, which is comprised of endothelial cells (bound by tight junctions), pericytes, microglia, astrocytes, and neurons (Bennett et al. 2019; Hawkins and Davis 2005) (Fig. 5). The neurovascular unit provides a structural barrier and metabolic support to the CNS. Neural interface devices that target the CNS compromise the BBB during the implant procedure (Moxon et al. 2009). These include electrodes for DBS, intracortical recording and stimulation, and intraspinal microstimulation. Initial electrode insertion causes mechanical damage to neurons, myelin, and vasculature (Jorfi et al. 2015; Mirkiani et al. 2024). This disruption of the BBB and vasculature causes systemic immune cells such as macrophages to infiltrate the implant site (Fig. 6A). Macrophages can remain at the implant site chronically (McConnell et al. 2009). Factors released by immune cells can oxidize electrode surfaces and even create cracks in lead insulation (J. M. Anderson et al. 2008a, b; Kao et al. 1994). Red blood cells also enter the implant site, increasing iron levels in the neural tissue (Wang 2010). Hemolysis after bleeding releases more iron into the implant site. Iron is dangerous in neural tissue because it causes Fenton reactions (Goldstein et al. 2003), which lead to excitotoxicity (Regan and Panter 1996), as well as the formation of reactive oxygen species that cause oxidative stress and neuronal degeneration, injury, and death (Goldstein et al. 2003; Regan and Panter 1996; Ward et al. 2014).

In the sub-acute phase of the reaction to implants in the CNS, resident microglia become activated (Fig. 6B); they transition from a dormant surveyor cell to a phagocytic cell, cleaning cellular debris and producing proteins for iron storage (Dheen et al. 2007; McCarthy et al. 2018; Polikov et al. 2005). The activated microglia release pro-inflammatory factors such as reactive oxygen species, nitric oxide, and reactive nitrogen species, which contribute further to localized neural degeneration and death (Biran et al. 2005; Block et al. 2007), additional damage the BBB (Bennett et al. 2018), and degrade electrode materials (Takmakov et al. 2015). The pro-inflammatory factors released by the activated microglia lead to the migration and activation of even more microglia from the surrounding parenchyma (Hermann and Capadona 2018). Activated microglia typically stay within a 20–35 µm thick perimeter around the electrode in both brain and spinal cord implants (Ersen et al. 2015; McConnell et al. 2009), leading to an increased impedance and reduced efficacy of the implant. A leaky BBB allows further influx of macrophages from the circulatory system to infiltrate the implant site (Ravikumar et al. 2014). Macrophages are activated and release or initiate the synthesis of inflammatory factors and mediators such as reactive oxygen species, proteases, tumour necrosis factor (TNF)-α, interleukin (IL)-1-β, enzymes, acids, and nitric oxide in an attempt to and seek to destroy the implant (Bennett et al. 2019; Tresco and Winslow 2011). These factors released by macrophages are also neurotoxic and can induce tissue damage in the region surrounding the implant. Activated macrophages have been observed at the implant site into the chronic stage of the tissue response. When electrodes are implanted near a major blood vessel without damaging it, astrocytes and microglia activity is still increased (Kozai et al. 2012b). The implantation of microelectrodes leads to an up-regulation of chondroitin sulfate proteoglycans (CSPGs) at the electrode-tissue interface (Zhong and Bellamkonda 2007). CSPGs are a component of the extracellular matrix and inhibit neural regeneration in the tissue adjacent to the implant site (Hynds and Snow 1999; Kuffler et al. 2009), contributing to an increased impedance of the electrode-tissue interface, which reduces the signal-to-noise ratio for recording electrodes and limits the electric field for stimulating electrodes.

In the chronic phase of the tissue response to an implant in the CNS, which occurs within days to weeks, microglia are less prevalent and astrocytes become hypertrophic and migrate to the implant site (Hermann and Capadona 2018) (Fig. 6C). Astrocytes isolate the implant from the surrounding tissue by forming a fibrous capsule around the electrode, known as the glial scar (Biran et al. 2005; Turner et al. 1999). Electrode encapsulation is further supported by fibroblasts from the meninges, specifically the pia mater (Barrese et al. 2013). Gliosis also occurs along the electrode tract, but is similar for stimulating and non-stimulating electrodes in the spinal cord (Bamford et al. 2010). Electrical stimulation has been shown to dampen the tissue response to DBS electrodes (Lempka et al. 2009), but is dependent on stimulation parameters. The collective effects of the glial scar reduce the number of neurons via neuronal degeneration, ultimately reducing the signal-to-noise ratio and electric field strength.

Non-penetrating neural interfaces in the CNS also evoke a tissue response. Micro-ECoG arrays implanted on rat cortices become encapsulated with fibrous scar tissue from the meninges (Schendel et al. 2014). Retinal implants typically do not compromise the blood-retinal barrier and therefore are not susceptible to the negative effects of the systemic immune system and iron. However, retinal microglia respond to injury within minutes (Eter et al. 2008; Lee et al. 2008) and migrate to the implant upon contact (Opie et al. 2012); the rest of the typical CNS tissue response follows. Electrical stimulation on the cortical surface can evoke a tissue response, where the extent of fibrosis is proportional to the charge density (Brown et al. 1977; Dauth et al. 1977). Conversely, recording electrodes implanted endovascularly in the brain become integrated into the blood vessel wall over time, avoiding the tissue response of the CNS (Opie et al. 2017; Oxley et al. 2016).

#### Peripheral nervous system tissue response

The peripheral nervous system (PNS) includes the spinal and cranial nerves. The auditory nerve becomes a peripheral nerve as it enters the cochlea; cochlear implants activate auditory neurons via an electric field surrounding the electrodes and transmitted in the perilymph (i.e., cochlear implants do not directly interface with neurons). The tissue response in the cochlea also involves the same cells (macrophages) and reactions as the PNS (Foggia et al. 2019). The exception is a severe and abnormal tissue response to a cochlear implant: neo-ossification (Dalrymple 2021; Foggia et al. 2019; Nadol et al. 2014, 2001). Neo-ossification, or new bone growth, occurs in the cochlea due to severe insertion trauma (Bas et al. 2015). Neo-ossification can exacerbate the loss of residual hearing and reduce the efficacy of cochlear implants by forming a resistive barrier between the electrodes and the auditory neurons (Foggia et al. 2019).

Implanting a device in the periphery causes trauma and vasculature damage. Excessive bleeding can lead to a hematoma, which can increase the risk of infection. The initial response to a foreign body in the periphery is the adsorption of blood plasma proteins, including albumin, fibrinogen, fibronectin, kininogen, complement, γ-globulin, and vitronectin, onto the surface of the implant (J. M. Anderson et al. 2008a, b; Klopfleisch and Jung 2017). These proteins form a provisional matrix around the implant, which then develops into a thrombus and eventually into a fibrin clot.

**Fig. 7:**
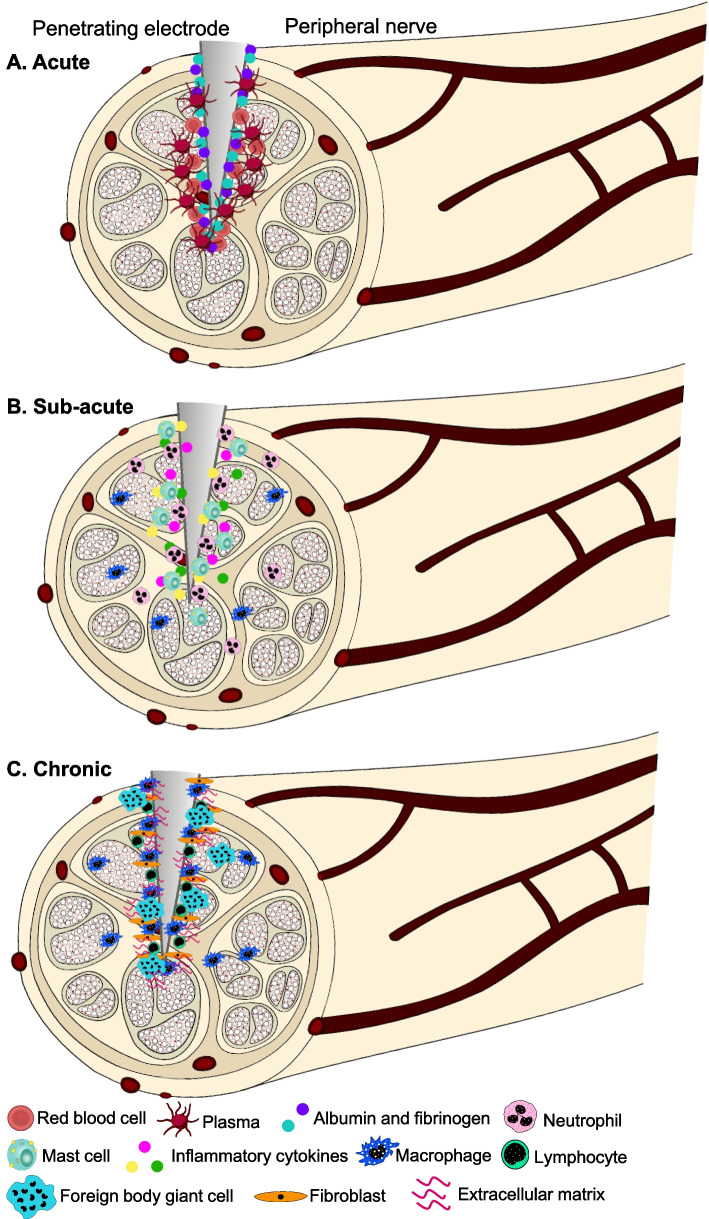
Acute, sub-acute, and chronic tissue response to implanted neural interfaces in the peripheral nervous system

The acute inflammatory response to implanted devices includes the infiltration of neutrophils (J. M. Anderson et al. 2008a, b; Bas et al. 2015; Fig. 7A). Neutrophils phagocytose debris and bacteria and enter the implant site within a few hours (Kastellorizios et al. 2015). In the sub-acute phase, mast cells enter the implant site and degranulate, releasing histamine and inflammatory cytokines (Tang et al. 1998; Fig. 7B). Histamine is also important for the recruitment of phagocytes such as neutrophils and macrophages to the implant site.

The presence of macrophages and lymphocytes mark the chronic phase of the tissue response (Fig. 7C). Macrophages and lymphocytes surround and adhere to the implant (Foggia et al. 2019; Klopfleisch and Jung 2017). Macrophages release several chemoattractants that further recruit more macrophages to the implant site (Broughton et al. 2006). As in the CNS tissue response, macrophages release factors and mediators that attempt degrade the implant. If the foreign body remains, macrophages can fuse together to form foreign body giant cells (FBGCs) (Klopfleisch and Jung 2017; Sheikh et al. 2015). FBGCs, if present, continue to release factors in an effort to completely break down the implant (Foggia et al. 2019; Henson 1971). In response to macrophage activation and the presence of FBGCs, fibroblasts and endothelial cells migrate to the implant site and proliferate (Foggia et al. 2019; Klopfleisch and Jung 2017). They release extracellular matrix proteins such as collagen to form granulation tissue around the implant. Over time, the granulation tissue forms an irreversible fibrous capsule around the implant (Lee et al. 2016), increasing the impedance of the electrode-tissue interface.

Nerve implants that do not penetrate the epineurium, such as cuff electrodes on the peripheral and vagus nerves, also induce a foreign body response, albeit milder than for penetrating electrodes (Rodríguez et al. 2000). A risk with cuff electrodes is that they are too tight around the nerve, inducing a compression injury that could lead to permanent nerve damage distal to the implant (Grill and Mortimer 2000). Additionally, the stiffness of the cuff electrode can influence the tissue response, with a greater tissue response occurring with more stiff electrodes (Stiller et al. 2019). A spiral cuff is used in clinical applications to reduce the risk of nerve compression and allows for short-term swelling of the nerve following initial implantation (Grill and Mortimer 2000; Naples et al. 1988). The tissue response to a cuff electrode includes encapsulation with macrophages, fibroblasts, and collagen, as well as focal regions with perineurial thickening, fibrosis of the endoneurium, thinning myelin, and reduced axonal density (Grill and Mortimer 2000; Payne et al. 2019).

#### Device encapsulation

Barring a continued inflammatory response and device failure, the final stage of the tissue response in both the central and peripheral nervous systems is device encapsulation. Encapsulation occurs because the immune cells are unable to digest the implant; instead, a protective barrier is formed to separate the device from the surrounding healthy tissue. As a result, the impedance at the electrode-tissue interface is elevated, but is typically stable (Dalrymple et al. 2020a; Groothuis et al. 2014; Jeffery et al. 2014; Wilk et al. 2016; Williams et al. 2007; Xu et al. 1997). Device encapsulation complicates the removal or replacement of implants or implant components because the scar tissue can integrate strongly to the device and surrounding tissues (Merrill 2014). However, there are benefits to encapsulation, including physical stability of the implant and protection from macrophage-secreted factors (Jorfi et al. 2015).

### Micro- and macro-motion: all you had to do was stay

Migration of lead wires and implanted electrodes can occur if there is any tension on the lead wires, or in response to gravity (such as with SCS, DRG, or sacral stimulation electrodes) (Cameron 2004; Huygen et al. 2020; Lyons et al. 2004; Nanivadekar et al. 2023; Zbar 2014). Lead wire migration or even complete removal of electrodes can occur if the implant is not secured, leading to device failure. However, not all implanted devices require lead fixation; cochlear implants remain stable in the temporal bone and do not require additional fixation of the lead wires. Lead wire migration could possibly cause damage to nearby structures and warrant a revision surgery for removal or reimplantation of components. The physical stability provided by the tissue response can reduce the risk of lead migration and electrode removal, instead securing the devices in place. However, the tissue response around the implant can also increase stiffness of the lead wires and electrodes, compounding the risk of further tissue damage from movement. Tissue encapsulation around micro-implants, such as intracortical electrodes, can displace or extrude the implant, leading to a loss of neuronal recordings due to an increased distance from the electrode (Barrese et al. 2013; Rousche and Normann 1998).

Perpetual movement of an implanted neural interface relative to the tissue can evoke an ongoing tissue response. Movements can be divided into two categories: macro-motion and micromotion. Macro-motion is larger-scale movements of the implanted devices relative to the tissue. Examples include movements of the spinal cord relative to the spine, which could affect the mechanical and electrode stability of intraspinal electrodes (Toossi et al. 2017), or intramuscular or intrafascicular electrodes shifting relative to muscles during limb movements (Pena et al. 2017). Relative movements between the tethered fixation points of the implanted devices and the tissue can lead to lead wire tension and, in extreme cases, partial or complete removal of the implant (Biran et al. 2007; Kim et al. 2004). Furthermore, as mentioned above, repeated bending and tension on the lead wires can induce metal fatigue and fracture, ultimately causing lead wire breakage.

Micromotions are more subtle movements of the tissue relative to the implanted shank or electrodes. Micromotion can be caused by respiration, heartbeat, changes in blood pressure, changes in cerebrospinal fluid (CSF) pressure, and general movements within the interstitial space (Kozai et al. 2015; Mahajan et al. 2020). Tissue damage occurs during micromotion due to a mechanical mismatch between stiff shanks and electrodes and the soft tissue (Barrese et al. 2013; Goldstein and Salcman 1973; Groothuis et al. 2014; Subbaroyan et al. 2005). Micromotion can induce compression or shearing of surrounding neural tissue (Cheung 2007), inducing a more vigorous tissue response (Kim et al. 2004). If electrodes are sharp, micromotion produces the strongest tissue response and gliosis at the tips (Edell et al. 1992; Kozai et al. 2015; McCreery et al. 2010). The tips themselves undergo changes in their structure and function due to micromotion, including deterioration and recessing, which results in a loss of recording signal over time, and insulation cracks or peeling away from the shank, which can reduce the specificity of the recording (Kozai et al. 2015; Prasad et al. 2012). Micromotion of an implanted device in the spinal cord is more harmful than in the brain, likely due to relative size (Ersen et al. 2015).

Neuron degeneration and loss near the implant site has been extensively studied in the cortex due to the high failure rate of intracortical recording electrodes. Electrode insertion, the tissue response, and micromotion contribute to neuronal loss surrounding an implanted electrode (Biran et al. 2007, 2005; Jorfi et al. 2015; Moxon et al. 2009; Prasad et al. 2012), with the largest loss of neurons occurring shortly after the implantation of the electrode array (Winslow et al. 2010). Neuronal loss can continue for the duration of the implant due to local, chronic inflammation, inducing a focal neurodegenerative state (McConnell et al. 2009). Explanted intracortical electrodes can be surrounded by densely packed layers of activated microglia (Szarowski et al. 2003; Turner et al. 1999), with the density of microglia inversely correlated with the neuronal density surrounding the electrode (Biran et al. 2005). In both stimulating and recording electrodes, there is more gliosis and neuronal loss near the electrode tip due to micromotion (Edell et al. 1992; Kozai et al. 2015; McCreery et al. 2021, 2010). As the radial distance away from the tip increases, so does the neuronal density (McCreery et al. 2010). There are also fewer and altered synapses adjacent to the glial scar (Schultz and Willey 1976). Reduced neuronal density near a microelectrode is detrimental to single unit recordings in particular, because neurons need to be within 130 µm of the recording site to be identified (Polikov et al. 2005). It has been suggested that neurons migrate away from the implant site (Collias and Manuelidis 1957; Liu et al. 1999); however, another study failed to find an increased neuronal density further away from the implant site, suggesting that neuronal loss, rather than migration occurs (Biran et al. 2005).

## End game: clinical implications of device failure

Implanted neural interfaces are designed to treat neurological disorders. When these devices fail, either technologically or biologically, there are clinical consequences. These clinical consequences can include the loss of therapeutic efficacy and return of symptoms or dysfunction, but can also include new clinical complications that are a direct result of the implanted device failure, including off-target effects, infection, and tissue damage. Complications, or adverse events, are reported to the United States Food and Drug Administration (FDA) and classified as either Serious Adverse Events (SAEs) and Adverse Events (AEs) (FDA 2024). However, the lines between SAEs and AEs are often blurry and inconsistently reported in literature and by hospitals (Barlas 2017; Gagliardi et al. 2018; Tilz et al. 2024). Medical device failures are documented in the Manufacturer and User Facility Device Experience (MAUDE) Database (Health 2024). However, this includes all medical devices, not just implanted neural interfaces. If device malfunction occurs repeatedly, it can lead to recalls by the FDA (FDA 2025a, 2025b, 2023, 2019). Table 2 summarizes the reported rates of clinical complications related to the failure of implanted neural interfaces.

**Table 2:** Reported rates of clinical consequences of implanted neural interface device failure. References: (Branco et al. 2023; Chapman et al. 2024; da Cruz et al. 2016; Daschner et al. 2017; Deng et al. 2006; Eldabe et al. 2016; FDA 2023, 2020; Force and da Silva 2017; Garg and Wang 2023; Goudman et al. 2024; Hines et al. 2022; Hoffmann et al. 2023; Horan et al. 2020; Ilfeld et al. 2017; Kahlow and Olivecrona 2013; Lander et al. 2020; Meng et al. 2018; Mitchell et al. 2023; Moman et al. 2021; Morishita et al. 2017; Mostafa and El Fiky 2024; Olson et al. 2023; Orlando and Cruz 2024; Pepper et al. 2013; Rizzo et al. 2020; Rolston et al. 2016; Rueb et al. 2020; Shibata et al. 2015; Sivanesan et al. 2019; Spindler et al. 2023; Toffa et al. 2020; Triolo et al. 2018; Vanloon et al. 2025; White-Dzuro et al. 2016)

*Name*	*Complication(s) and Rate(s)*
Deep brain stimulation (DBS)	Hardware-related infections (4%); readjustment of lead position (2.7%); lead fracture (1.4%); lead migration (12.3%); pneunomia (2.3%); hematoma (1.4%); intracranial bleeding (6.1%); pulmonary embolism (0.6%); death (0.2–0.32%)
Electrocorticography (ECoG)	Surgical site infection (4%); hematoma (1–7.3%); infection from subdural placement (2.4–15.6%); seizure (1–41%); deep vein thrombosis (2.3%); sepsis (< 1%); death (< 1%)
Depth electrodes	Surgical site infection (2.4–14.9%)
Stentrode endovascular electrode array	Hematoma at insertion site (1/4 participants)
Spinal cord stimulation (SCS)	Lead migration (3.07–9.97%); lead explant (2.02%); implanted pulse generator explant (2.67%); infection (3.4–10%); hematoma (0.81%); device malfunction (27.1%); spinal cord injury (0.42%); death (0.47%)
Dorsal root ganglion stimulation (DRGS)	Trial lead infection (1,03%); implant infection (4.8%); revision infection (3.85%); lead migration (0.7–9.1%); lead fracture (6%); lead migration (6%); lead defects (39%); revision (29%); fragments left following lead removal (12%); explant (12%); permanent nerve damage during replacement procedure (9.1%)
Cochlear implant	Surgical site infection (1.4–3.2%); hematoma or seroma (1.3–2.6%); major infection and necrosis (2.3%); device fault (0.5%); electrode extrusion (2.6%); permanent facial palsy (0.09%)
Retinal prosthesis	Revision surgery (3.4%); conjunctival erosion (6.2%); retinal detachment (6.7%); infection (16.7%)
Vagus nerve stimulation	Surgical site infection (2.6–3.5%); hematoma (1.9%); lead fracture (3–11.9%); lead disconnection (0.2–2.5%); stimulator malfunction (1.4%); battery displacement (0.2%); persistent vocal cord palsy (0.7%); deep infection requiring explant (3.5%); explant due to implanted pulse generator dysfunction (4–16.8%)
Peripheral nerve stimulation	Infection (0.1–0.7%); electrode failure (2–10%); lead fracture (6.25%)
Sacral stimulation	Infection (1.6–6.6%); seroma or hematoma (3%); pocket revision due to infection (14.6%); lead wire breakage (7.5%); lead fragments left behind following breakage (6%); lead migration (2.1%); battery depletion requiring reoperation (1.7–39%); lead revision (13–18%); explant (4–24%)

When a complication arises from failure of an implanted neural interface, it is important to understand how severe the complication is, so that the treating clinicians can determine the most appropriate treatment. There is a need for a clear guide to aid clinical decision making according to the severity of the failure. In Table 3, we have created such a guide by adapting the Clavien-Dindo grading system for surgical complications (Dindo et al. 2004). The examples provided were collected from the former sections, as well as from the troubleshooting algorithm developed by (Zbar 2014).

**Table 3:** Grading system developed for the severity of clinical complications resulting from implanted neural interface failure, including examples. This was modified from the Clavien-Dindo grading system for surgical complications

*Grade*	*Description*	*Examples*
I	Minor loss of efficacy or complication requiring no surgical or pharmacological intervention	Reprogramming, warm compress for edema, imaging
II	Complications requiring pharmacological intervention	Antibiotics or steroids for infection or edema
IIa	Single treatment	
IIb	Repeated treatment	
III	Complications requiring outpatient surgical intervention	Draining a hematoma or debriding skin necrosis
IV	Temporary loss of function or disability	Rehabilitation for nerve compression
V	Complications requiring inpatient surgical intervention	Revision, removal, or replacement of part or all of device
Va	Intervention not under general anesthesia	
Vb	Intervention under general anesthesia	
VI	Permanent loss of function or disability	Ongoing rehabilitation for paralysis, treatment of seizures
VII	Life-threatening complications requiring intensive care management	Sepsis from infection
VIIa	Single organ dysfunction	
VIIb	Multi-organ dysfunction	
VIII	Death related to the complication	Sepsis from infection

## Long live: chronic testing to improve device longevity

Often, the cause and effect of failure modes of implanted neural interfaces cannot be delineated between technological and biological factors. Additionally, a combination of failure modes can occur simultaneously. Therefore, redundancy and improved manufacturing processes are necessary to ensure reliability of implanted neural interfaces for the lifetime of the device user. Reliability is determined via the characterization of the technological and biological failure modes. This process typically and should entail benchtop testing, followed by acute and most importantly, chronic testing of implanted devices (Dalrymple 2021; Henderson et al. 2006; Pena et al. 2017; Shepherd et al. 2018). Computational modeling can also be used to predict and identify failure modes of implanted devices (Henderson et al. 2006; Jorfi et al. 2015; Subbaroyan et al. 2005). With in vivo testing, it is important to have clinically-relevant animal models for testing novel implanted neural interface systems. It is equally important to use electrode and implant assemblies that closely resemble the form factor and materials used in the proposed implant.

**Fig. 8:**
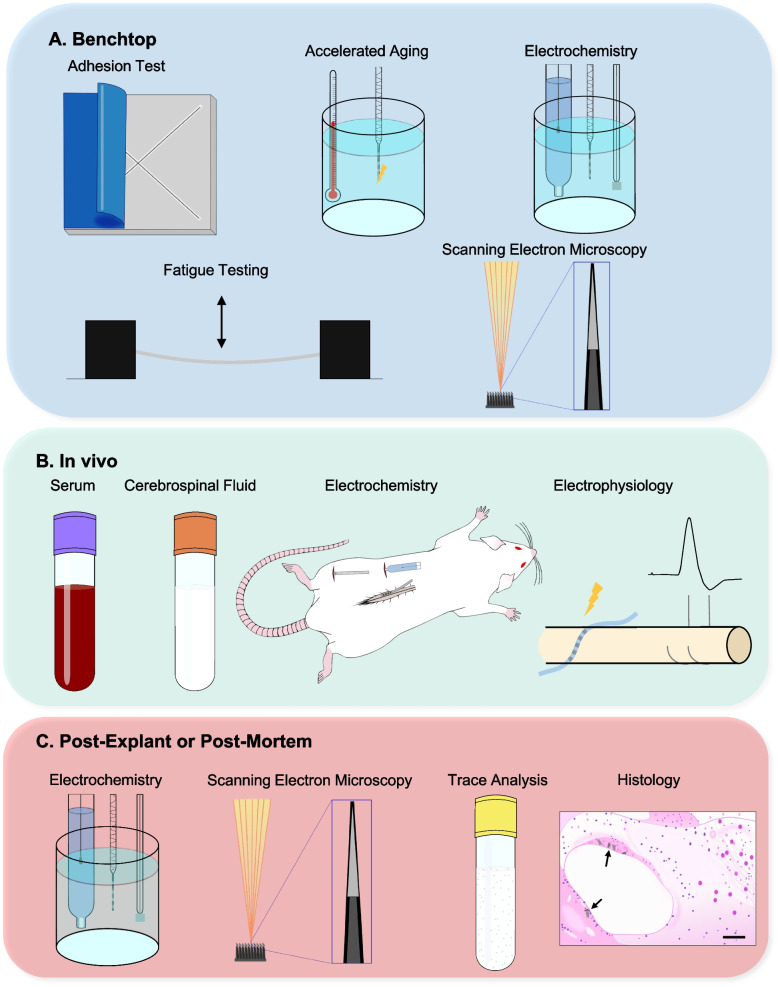
Overview of testing methods used to evaluate implanted neural interface systems. Benchtop methods include the adhesion test of the electrode material, accelerated aging of the implanted portion of the device, electrochemical measures such as CSC, CIL, EIS, and common-ground impedance, fatigue testing of lead wires, and SEM of the electrode surface. In vivo testing includes testing serum and CSF samples, electrochemical measures, and electrophysiology of evoked responses. Post-explant testing includes electrochemical measures on the explanted electrodes and SEM of the electrode surface. Post-mortem analysis includes trace analysis and histological examination of tissues

Several testing methods can be used to minimize or eliminate failure modes, or, if not out of the woods, can be used to study the mechanisms of failure and monitor the implant (Fig. 8). Lead wires are prone to breakage; therefore, extensive benchtop fatigue testing is necessary prior to implantation. Fatigue testing is an accelerated process that entails repeatedly bending lead wires until lead breakage or cracks in the insulation occur (Harris et al. 2016; Pena et al. 2017; Fig. 8A). Neural interfaces that have been implanted in people, such as DBS (Jiang et al. 2015), SCS (Henderson et al. 2006), and LIFEs (Pena et al. 2017) have reports characterizing their benchtop fatigue testing. The American Society for Testing Materials (ASTM) International has a standardized protocol for fatigue testing (ASTM 2020).

Coatings on electrode surfaces are at risk of delaminating; therefore, their adhesion must be tested prior to active in vitro or in vivo studies. The adhesion-by-tape test (ASTM 2022) is a simple yet effective method for testing the adhesion of electrode coatings (ASTM 2022; Dalrymple et al. 2019; Green et al. 2012; Fig. 8A). Coating material loss can be quantified following inspection using scanning electron microscopy (SEM; Čvančara et al. 2020; Dalrymple et al. 2019; Green et al. 2012; Fig. 8A,C). SEM can also be used to visualize damage to insulation or electrode tips, such as cracking, peeling, corrosion, or breakage (Prasad et al. 2012).

Accelerated aging is a benchtop process whereby electrodes are housed (passive) and/or stimulated continuously (active) in a saline-like solution at an elevated temperature (body temperature or higher; Fig. 8A). Accelerated aging protocols mimic the physiological environment but on a faster timescale (ASTM 2021; Hukins et al. 2008). The saline-like solution can be comprised of saline, phosphate-buffered saline (PBS) (Noller et al. 2019), artificial CSF (Vara and Collazos-Castro 2019), contain proteins such as bovine serum albumin, or contain reactive chemicals such as hydrogen peroxide, which mimics reactive oxygen species (Street et al. 2018; Takmakov et al. 2015). Accelerated aging protocols have been applied to many types of implanted neural interfaces, both clinically available devices and prototypes with novel electrode materials or coatings, including but not limited to functional neuromuscular stimulation devices (Smith et al. 1987), intracortical electrodes (Patrick et al. 2011; Street et al. 2018; Takmakov et al. 2015; Venkatraman et al. 2011), floating microelectrode arrays (Bredeson et al. 2013), cochlear electrodes (Dalrymple et al. 2019), and retinal prostheses (Lemoine et al. 2020). Accelerated aging can also be used to test novel hermetic packaging technologies (Nagarkar et al. 2017). Following the accelerated aging protocol, the electrode surface is often imaged using SEM to inspect for corrosion of the electrode surface or delamination of coatings, the solution is examined using mass spectroscopy for particulates of the electrode or coating material, the device is checked for open or short circuits, and/or the electrodes are tested using various electrochemical measures (Dalrymple et al. 2019).

Electrochemical measures describe the safety and effectiveness of an electrode to conduct and/or deliver charge at the electrode-tissue interface (Fig. 8A-C). Electrochemical measurements can be acquired benchtop using three electrodes: the working, reference, and counter electrodes in a saline-like solution (Cisnal et al. 2018; Cogan 2008; Dalrymple et al. 2019) or in vivo (Lempka et al. 2009; Shepherd et al. 2021). Charge storage capacity (CSC) is the amount of charge that can be stored in reversible reactions, i.e. without exceeding the water window (Merrill et al. 2005). The CSC is measured using cyclic voltammetry, where the electrode potential between the working and counter electrode is slowly cycled between the water window limits (Cisnal et al. 2018; Cogan 2008). The CSC depends on the electrode geometric surface area, material, electrolyte composition, and waveform parameters. It is desirable to have a large CSC such that more charge can be injected safely into the tissue to excite neurons. The charge injection limit (CIL) is the maximum amount of charge that can be injected into the tissue in reversible reactions during a stimulation pulse (Cisnal et al. 2018; Dalrymple 2021). The CIL is determined using voltage transients, where biphasic, charge-balanced, cathodic first pulses are delivered through the electrode at a constant pulse width and increasing current amplitudes (Lee et al. 2016). Impedance is typically measured using one oftwo methods: common ground impedance and electrochemical impedance spectroscopy (EIS). Common ground impedance entails measuring the voltage resulting from a small current stimulus and calculating the corresponding resistance using Ohm’s law (Shepherd et al. 2021). For optimal recording performance and high electrode yield, it is best if the electrode impedance is in the 2 to 150 kΩ range (Chen et al. 2022; Fu and Rutishauser 2025; Prasad et al. 2012); however, these values may vary for different applications. EIS produces a more comprehensive measure of impedance across a range of frequencies (Cogan 2008). EIS magnitude and phase values can be used to generate an equivalent circuit model, of which there are several types (Lempka et al. 2009; Lisdat and Schäfer 2008; Shepherd et al. 2021; Wei and Grill 2009). The components of the equivalent circuit model indicate both electrode and tissue behaviour.

Acute and/or chronic in vivo testing in animal models should follow benchtop testing to ensure the implanted devices can survive a more realistic environment (Fig. 8B). Much of the time, chronic in vivo testing is performed over a period of months, but can be executed for years in larger animal models (Barrese et al. 2013; Chestek et al. 2011; Christensen et al. 2014; Grill and Mortimer 2000; Jeong et al. 2015; Kane et al. 2013; Kozai et al. 2015; Lago et al. 2007; Nayagam et al. 2014; Opie et al. 2018; Oxley et al. 2016; Payne et al. 2018; Rodríguez et al. 2000; Sahasrabuddhe et al. 2021; Shepherd et al. 2021; Stock et al. 1979). Longer durations of chronic in vivo testing provide valuable insight into the device performance and tissue reaction to implants over a time-frame that more closely matches the duration in human implementation. Furthermore, the tissue response after several months is expected to be stable, entering the device encapsulation stage described above. However, long-duration chronic in vivo testing is costly, and investigators must balance resource availability with the gain of information from longer duration implants.

The location, size of the implant, and electrode geometry should scale to the animal model for the most accurate testing for the proposed clinical application. During and following the chronic implantation period, the implanted devices are characterized by how well they function and whether or not they maintained their physical integrity. For example, electrochemical measures can be used to track changes at the electrode-tissue interface over time (Abidian et al. 2010; Dalrymple et al. 2020b, 2020a; Jeong et al. 2015; Kane et al. 2013; Opie et al. 2016; Shepherd et al. 2021). Electrode corrosion or metal dissolution causes pitting on the electrode surface, increasing the surface area (Prasad et al. 2012; Shepherd et al. 2021). This increase in surface area results in an increased CSC, and can also reduce the impedance (Dalrymple et al. 2020b, 2020a; Merrill et al. 2005; Shepherd et al. 2021).

Insulation damage can result in a decreased impedance, due to an increased surface area of the conductive electrode (Prasad et al. 2014). Daily fluctuations in impedance can occur, likely also influenced by the tissue response to the implant, transient bleeding, and edema (Groothuis et al. 2014; Prasad et al. 2012). During current-controlled stimulation, a higher electrode impedance demands more power from the pulse generator because an increased stimulation amplitude is required to excite the same neurons (Butson et al. 2006). Continued increases in stimulation amplitude to maintain efficacy has been reported for DBS (Krack et al. 2002; Yamamoto et al. 2004).

For devices that transfer power wirelessly through the skin, such as cochlear implants, power transmission is limited by the wireless components and safety standards. Therefore, an increased power demand due to high electrode impedances may not be possible. While recording neural activity, the impedance can greatly impact the signal-to-noise ratio (Chen et al. 2022; Chu et al. 2012; Chung et al. 2015; Groothuis et al. 2014); a high impedance (> 1.5 MΩ) reduces the yield of single units recorded (Prasad et al. 2014). Periodic electrochemical assessment can inform on the state of the electrode-tissue interface and be used to explain changes in required stimulation amplitude to be effective. Characterizing chronic implants in vivo can reveal challenges that were not identified in benchtop or acute testing, especially those related to the tissue response or the delamination of electrode coatings (Abidian et al. 2010; Čvančara et al. 2020; Dalrymple et al. 2020b, 2020b; Green et al. 2012). Sometimes, the results of the chronic in vivo testing require a change in design, and begin the testing again, to ensure optimal biocompatibility and longevity. Therefore, these chronic in vivo studies must be performed to ensure that there are no surprises come time to translate these implants to clinical application.

Several electrophysiological measures can be used to monitor the implanted neural interface and how well it is interacting with neurons (Fig. 8B). In general, a decaying or loss of signal from recording neural interfaces can easily be measured over time, such as intramuscular EMG electrodes (DeMichele et al. 2013), ECoG arrays (Baek et al. 2014), or intraspinal electrodes (Greenspon et al. 2019). For stimulating electrodes, either the electrodes need to be connected to a recording device such that single units or local field potentials can be recorded through the stimulating electrodes, or recording electrodes are placed elsewhere along the neuraxis to measure an evoked response (McCreery et al. 2004; Prasad et al. 2012). Examples of evoked responses include evoked auditory brainstem responses (EABRs) elicited by stimulation through cochlear implants (Dalrymple et al. 2020a, 2020b; Shepherd et al. 2020), or evoked compound action potentials (ECAPs), which can be evoked by stimulating the DRG, vagus nerve, spinal cord, and periphery, and recorded from the peripheral nerves, vagus nerve, or spinal cord (Calvert et al. 2022; Dalrymple et al. 2021; Fisher et al. 2014; Payne et al. 2020; Shulgach et al. 2021; Ting et al. 2024). Chronic animal studies should monitor the natural and electrically-evoked neural activity longitudinally to ensure that the target neural population is being recorded/stimulated. In humans, patients may be able to report a response. For example, patients with a cochlear implant can report whether they can hear during stimulation, or retinal prosthesis users can report seeing phosphenes.

Blood serum and CSF samples can be extracted and analyzed throughout the duration of an implant to monitor the inflammatory response (Prasad et al. 2012; Fig. 8B). For example, phosphorylated neurofilament heavy subunit (pNF-H) is a biomarker for axonal injury that can be detected in both blood and CSF in response to ongoing axonal damage (K. J. Anderson et al. 2008a, b; Prasad et al. 2012; Shaw et al. 2005). Sustained and fluctuating elevated levels of pNF-H has been found following chronic implantation of intracortical electrodes in rats, indicating ongoing axonal damage (Prasad et al. 2012). Additionally, cytokine biomarkers that have been identified in CSF and serum samples in response to a spinal cord injury, such as IL−6, IL-8, monocyte chemoattractant protein (MCP)−1, tau, glial-expressed protein S100β, and glial fibrillary acidic protein (GFAP) (Kwon et al. 2010), may be useful to determine ongoing inflammation in response to electrodes implanted in the spinal cord. At the conclusion of the implant testing duration, tissue surrounding the implant as well as organs responsible for filtering toxins, such as the kidneys and liver, can be tested using trace analysis for metal or polymer particulates that may have originated from the electrodes (Shepherd et al. 2021; Fig. 8C). Furthermore, the tissue surrounding the implant can be excised, sectioned, and examined histologically for the presence and activation of immune cells (Dalrymple et al. 2020a, 2020b; McCreery et al. 2010; Nayagam et al. 2014; Schendel et al. 2014; Fig. 8C).

When neural interfaces are implanted into people, they can be monitored over the duration of the implant using the aforementioned methods, especially impedance or evoked response testing (Fisher et al. 2009). Early feasibility and first-in-human trials aim to assess the safety and efficacy of implanted neural interfaces (Ayton et al. 2014; Bergey et al. 2015; Čvančara et al. 2020; Hochberg et al. 2006; Kilgore et al. 2003; Mitchell et al. 2023). In the rare instances that implanted electrodes are explanted, the electrode surface and electrochemical behaviour can be characterized (Woeppel et al. 2021; Fig. 8C). Otherwise, investigating how the implanted neural interfaces interact with the tissue or inspecting the electrodes for corrosion is done post-mortem (Haberler et al. 2000; Moss et al. 2004; Nadol et al. 2014; O’Malley et al. 2017; Szymanski et al. 2021; Towle et al. 2020). Post-mortem examination of tissue is extremely informative because it reveals the tissue response and electrode integrity after lifetime use of the implant (Fig. 8C).

## Wildest dreams: the future of implanted neural interfaces

The therapeutic successes of many implanted neural interfaces have sparked a dynamic industry (Weber 2020) as well as many thematic funding opportunities, including ElectRx, BG + , ReNet, N3, NESD, RAM, SUBNETS, TNT, and HAPTIX by the Defense Advanced Research Projects Agency (DARPA) and other major funding agencies such as the National Institutes of Health (NIH) and Department of Defense (DoD) in the United States of America. With technological advances in nanoengineering, materials science, electromagnetism, and optogenetics, the future of implanted neural interfaces is bigger than the whole sky, but not untouchable.

Through chronic in vivo experiments, failure modes of implanted neural interfaces can be identified, and innovative solutions can be applied to ameliorate them. As described, many different animal models have been used for preclinical testing of implanted neural interfaces. Mouse models for implanted neural interfaces open many doors of investigation. For example, to better understand the specific genes, enzymes, and cellular signalling pathways that may influence the performance of the neural interfaces, transgenic mouse models have been developed (Bedell et al. 2018a, 2018b; Hermann et al. 2018b, 2018a; Kozai et al. 2014b). Furthermore, 2-photon microscopy can be used to perform live imaging of the mouse brain, particularly to study the live tissue response to intracortical electrodes (Kozai et al. 2012b, 2016). Mouse models also enable the use of optogenetics, which can be used to locate specific cell types responsible for the recorded electrophysiological behaviour (Anikeeva et al. 2011; Park et al. 2017; Pashaie et al. 2014). Mouse models for studying implanted neural interfaces can be challenging, especially with the size limitations; however, a recent study showed that the strain on cortical tissue from a microelectrode implant was no different in a mouse compared to a rat model (Mahajan et al. 2020).

Surgical approaches and implantation techniques can be modified to invoke less trauma to the tissue. For example, delivering electrodes by injecting them through a syringe, such as with the Injectrode (Dalrymple et al. 2021; Trevathan et al. 2019) or ultra-flexible mesh electronics (Fu et al. 2017; Hong et al. 2018; Liu et al. 2015), can both quicken the implant procedure time and reduce the trauma to surrounding tissues. Additionally, updated methods for securing lead wires can be improved to reduce lead migration, cracks in insulation, and infections, as has been demonstrated for DBS implants (White-Dzuro et al. 2016). Improved materials, such as alginate hydrogel, have improved the seal in the dura mater following electrode implantation (Nunamaker and Kipke 2010). Intracortical and ECoG recordings require the removal of a portion of the skull to implant the electrodes and depth electrodes require small holes to be drilled into the skull for insertion. An alternative recording device, the Stentrode, is implanted endovascularly near the motor cortex, reducing both the surgical trauma and avoiding the tissue response from the brain (Oxley et al. 2020, 2016). However, one downside to techniques such as the Stentrode or ECoG are that the electrodes are at a greater distance from the neurons; therefore, they record local field potentials rather than individual neuron spikes. This may limit the specificity and degrees of freedom in the recordings of these techniques, but new signal processing methods and decoders have demonstrated the utility of these technologies (Forsyth et al. 2019; Luo et al. 2022; Volkova et al. 2019).

Wireless communication and power transfer between external and internal components or between the stimulator and electrode can reduce the incidence of lead wire breakage. However, as reviewed above, wireless methods are not without limitations. Innovative methods to transfer data and/or power are being developed to mitigate issues related to heating, overlap requirements, transmission efficiency, and form factor (Robinson et al. 2024). These technologies leverage RF and inductive coupling, volume conduction, ultrasound, optics, and magnetoelectrics (Becerra-Fajardo et al. 2024; Benedict et al. 2022; Kim et al. 2023; Lee et al. 2021; Tawakol et al. 2024), including neural dust for recording from peripheral nerves (Seo et al. 2016). Additionally, battery-free technologies that harvest energy from the body are under development to facilitate distributed networks of implanted neural interfaces that do not require charging or battery replacement (reviewed in Nair et al. 2023).

Reducing the stiffness of hermetic packaging, lead wires, and electrodes has also been explored to reduce failure of implanted neural interfaces. Reducing the stiffness of implanted devices to more closely match that of the surrounding tissue leads to a reduced inflammatory response (He et al. 2020; Jorfi et al. 2015; Patel and Lieber 2019; Sohal et al. 2016). Flexible hermetic packaging made from silicone, polydimethylsiloxane (PDMS), parylene, polyimides, epoxies, polyurethanes, and liquid crystal polymers have been explored as an alternative to the conventional titanium packaging (Hassler et al. 2011; Jeong et al. 2015; Nagarkar et al. 2017; Rubehn et al. 2009). However, many of these polymeric materials are porous to water vapour and degrade under physiological conditions (Hassler et al. 2011; Traeger 1977).

Low stiffness materials have also been used for electrodes and arrays (Fekete and Pongrácz 2017). For example, electronic dura (e-dura) is an array capable of recording, electrical stimulation, and chemical injection, and has the same elasticity as the dura mater (Minev et al. 2015). Other flexible arrays for SCS have been developed and tested in rats (Hogan et al. 2021) and flexible microscale wires have been implanted in the brain of mice for recording (Yin et al. 2022). Flexible microelectrode arrays have been developed and tested in slug DRG (Sperry et al. 2018) as well as rodent brains (Harris et al. 2011b; Zhao et al. 2022). Flexible depth electrodes have been implanted in the brains of small and large animal models (Lee et al. 2024). Intrafascicular electrodes typically use stiff needles (Badia et al. 2011); more recent designs utilize microneedles embedded in soft silicone, resulting in stretchable and flexible intrafascicular electrodes for recording from peripheral nerves (Yan et al. 2022). Novel polymeric materials can be used for electrodes on peripheral nerve cuffs, enabling them to stretch with the cuff (Cuttaz et al. 2021). Arrays with multiple penetrating electrodes and flexible bases can reduce the relative motion of the electrodes (Khaled et al. 2013). Flexible electronics using nanotechnologies have been used for detecting biomarkers (Farsinezhad et al. 2013; Yan et al. 2021), intracellular recording and stimulation (Robinson et al. 2012), and intracortical recording (Zhao et al. 2019, 2017).

Coatings on electrodes and shanks can be used to improve acceptance. Conductive hydrogel coatings applied to cochlear implants (Dalrymple et al. 2020b), DBS electrodes (Hyakumura et al. 2021), and electrodes implanted in the auditory cortex (Kim et al. 2010) reduce the stiffness and impedance of the electrode. Hydrogel electrodes have also been used for flexible cuff electrodes around the cervical vagus nerve to allow for adjustments in diameter of the cuff (Horn et al. 2021). Mechanical insertion damage can be reduced by using less stiff electrode and shank materials; however, they need to be stiff enough to penetrate tissue but not too stiff that causes excessive damage. One solution to this problem is to use a stiff implant carrier that dissolves away, such as carboxymethyl cellulose (Gilgunn et al. 2012; Kozai et al. 2014a), resorbable polymers (Lewitus et al. 2011), or polyethylene glycol (Kato et al. 2006), leaving behind flexible electrodes. Another option is to use a stiff implant carrier that is removed, leaving the softer electrode behind (Ferro et al. 2018; Hanson et al. 2019; Kozai and Kipke 2009; Musk and Neuralink 2019; Williamson et al. 2015). Furthermore, temperature-sensitive and mechano-sensitive polymers that soften at physiological temperatures (Capadona et al. 2008; Hess et al. 2013; Ware et al. 2014) can reduce the loss of neuronal density near the device (Harris et al. 2011a) as well as reduce tissue deformation (Garcia-Sandoval et al. 2018). Similarly, shape memory polymers that soften in vivo have been explored to reduce the stiffness of neural implants (Sharp et al. 2006; Ware et al. 2014).

Novel electrode and array geometries that are porous or latticed have been designed with the intention of integrating the device with the tissue. For example, micro-ECoG arrays constructed with a mesh-like substrate showed a reduced thickness of meningeal tissue growth between the array and the neural tissue (Schendel et al. 2014). The holes in the mesh enabled revascularization of the tissue around the device. Several designs for intracortical probes with porous structures have been developed to allow neural, connective, and vascular tissues to grow through the pores instead of encapsulating the device (Kang et al. 2011; Seymour and Kipke 2007, 2006; Xie et al. 2015). Similar neurovascular integration has been achieved with a porous peripheral neural interfaces as well (Veith et al. 2021). However, removal of these integrated devices in the event of failure or infection may lead to excess tissue damage.

The acceptance of an implant by the surround tissue can be enhanced using coatings that are bioactive (Chapman et al. 2020; Klopfleisch and Jung 2017; Rousche et al. 2001). Bioactive refers to using coatings that control or calm down the intrinsic tissue response. Bioactive coatings may contain peptides that promote neurite outgrowth (Green et al. 2009) and reduce microglia activation and migration (Azemi et al. 2011; Sridar et al. 2017), reduce protein fouling (Golabchi et al. 2019; Kozai et al. 2012a; Rao et al. 2012), release anti-inflammatory agents (Gaire et al. 2018; Kim and Martin 2006; Krukiewicz et al. 2019; Wadhwa et al. 2006; Zhong and Bellamkonda 2007, 2005), prevent glial scar formation (He et al. 2006; Massia et al. 2004; Tien et al. 2013), catalyze reactive oxygen species (Potter-Baker et al. 2014), or release trophic factors to attenuate neural degeneration (Chikar et al. 2012; Kato et al. 2006). Neural interfaces can also host microfluidic systems for delivering factors that reduce the tissue response (Altuna et al. 2013; Frey et al. 2018; Takeuchi et al. 2005). Not all implants need to remain implanted forever and always; temporary monitoring of intracranial pressure or evoked potentials could be realized through resorbable biosensors (Kang et al. 2016). Resorbable biosensors make use of materials such as poly(lactic-co-glycolic acid) (PLGA), nanoporous silicon, magnesium foils, and silicon dioxide that undergo hydrolysis during implantation, dissolving after approximately four to five weeks (Gentile et al. 2014).

Stimulation safety limits ensure that the electrode polarization does not exceed the water window. However, the recommended stimulation safety limits were derived from a study that chronically implanted platinum electrodes that were stimulated over a few hours into brain tissue (McCreery et al. 1990). Because the central and peripheral nervous systems have different immune cells, hence different tissue responses to implanted devices, stimulation safety limits should be determined independently for each region of the body. For example, high charge stimulation (exceeding the stimulation safety limits) of the cochlea did not result in neuronal death (Shepherd et al. 2021) but did result in corrosion of platinum from the electrode, and platinum particulates in the tissue capsule. Therefore, new materials need to be developed that can tolerate high charge stimulation such that stimulation limits can be identified for all interface sites. Many new materials have been designed with the goal of reducing electrode impedance, which allows for more and smaller electrodes, and a wider stimulation range, which can improve selective activation of neurons (Ludwig et al. 2011). For example, high surface area materials such as reduced graphene oxide, conductive hydrogel, and electrodeposited Platinum-Iridium have been explored for reducing the impedance of cochlear electrodes (Dalrymple et al. 2020a, 2020b, 2019). Furthermore, many different Poly(3,4-ethylenedioxythiophene) (PEDOT) formulations have been developed and tested for intracortical electrodes (Ganji et al. 2018; Ludwig et al. 2006; Seymour et al. 2011; Venkatraman et al. 2011), intraspinal microstimulation (Vara and Collazos-Castro 2019), and peripheral nerve cuffs (Lee et al. 2016).

## The other side of the door: data storage and programming considerations

In addition to addressing the biological and hardware-related failure modes, there are software and data-related concerns that are important to consider in the development, optimization, and translation of implanted neural interfaces. Modern and future implanted neural interfaces seek to interface with more neurons, which demands more electrodes that are smaller and more selective in their recordings and/or activation. With this increased demand, the complexity of both processing recordings and delivering stimuli increases. More sophisticated programming methods are needed, beyond the simple input–output and closed-loop systems currently in use. Neurons can be more precisely activated during stimulation by improving both spatial and temporal targeting. Spatial methods manipulate the strength and shape of the electric field to specify which neurons are activated. The electric field can be adjusted by changing the stimulation amplitude, pulse width, and pulse train frequency, as is common with modern devices. Electrode size, number, configuration (e.g., multipolar), and current steering methods can shape the electric field to optimally and precisely activate neurons (Dumm et al. 2014; Mishra et al. 2023; Tebcherani et al. 2024). Typical stimulation methods activate neurons synchronously, which is not how neurons naturally fire. Neurons can be activated more closely to their natural firing patterns using biomimetic stimulation, which entails modulating both amplitude and frequency of stimulation pulses (Formento et al. 2020; Okorokova et al. 2018).

Strategies to control the timing or intensity of stimulation determine when and how many neurons are activated. While current clinical devices have relatively simple control strategies that are often open-loop, closed-loop control is become more common. Closed-loop methods use recorded signals (for example, ECAPs) to improve the effectiveness and efficiency of the stimulation method (Brooker et al. 2021; Kuo et al. 2018). Control algorithms can be made more personalized and powerful with the use of machine learning (Dalrymple et al. 2020c; Dalrymple and Mushahwar 2020; Desautels et al. 2015). Informative and real-time signals are required to inform control strategies, which can be achieved through onboard sensing and processing of biosignals, and neural decoding methods.

With the gathering of large amounts of neural data, security concerns arise, particularly with how the data are transferred and stored (Jiang et al. 2023; Maiseli et al. 2023). Cloud-based data storage and computing, as well as the use of AI-methods such as large language models to interpret data are growing in popularity and present concerns with personal health information. Methods for enhancing security and ensuring ethical data handling must continually adapt alongside rapid technological advancements. Moreover, policies governing the approval and regulation of implanted neural interfaces need constant updating to align with these evolving developments.

## Conclusions

Long story short, neural interfaces implanted throughout the body have demonstrated great success in treating a growing variety of conditions. Despite these successes, the longevity of implanted neural interface systems are impeded by mechanical, technological, and biological barriers. Mechanical and electronic failures can occur in any of the components of the implanted system. The immune response to an implanted neural interface consists of acute and chronic phases and differs between the central and peripheral nervous systems. Advances in material science and engineering are actively working to reduce the tissue response to implanted neural interfaces by reducing their size and stiffness as well as by using factors to reduce inflammation. Cycles of improving these devices and materials with chronic in vivo testing is needed to thoroughly test new systems prior to clinical translation to ensure their long-term biocompatibility for human implantation.

## Data Availability

No datasets were generated or analysed during the current study.
